# The heparan sulfate mimetic PG545 interferes with Wnt/β-catenin signaling and significantly suppresses pancreatic tumorigenesis alone and in combination with gemcitabine

**DOI:** 10.18632/oncotarget.3214

**Published:** 2014-12-31

**Authors:** Deok-Beom Jung, Miyong Yun, Eun-Ok Kim, Jaekwang Kim, Bonglee Kim, Ji Hoon Jung, Enfeng Wang, Debabrata Mukhopadhyay, Edward Hammond, Keith Dredge, Viji Shridhar, Sung-Hoon Kim

**Affiliations:** ^1^ College of Korean Medicine, Kyung Hee University, Seoul, South Korea; ^2^ Korean Medicine Clinical Trial Center, Kyung Hee University, Seoul, South Korea; ^3^ Department of Biochemistry and Molecular Biology, MN, USA; ^4^ Progen Pharmaceuticals Ltd, Brisbane, Queensland, Australia; ^5^ Experimental Pathology, Mayo Clinic College of Medicine, Rochester, MN, USA

**Keywords:** PG545, Wnt/β-catenin, pancreatic cancer, heparan sulfate mimetic, gemcitabine

## Abstract

The heparan sulfate mimetic PG545 has been shown to exert anti-angiogenic and anti-metastatic activity *in vitro* and *in vivo* cancer models. Although much of this activity has been attributed to inhibition of heparanase and heparan sulfate-binding growth factors, it was hypothesized that PG545 may additionally disrupt Wnt signaling, an important pathway underlying the malignancy of pancreatic cancer. We show that PG545, by directly interacting with Wnt3a and Wnt7a, inhibits Wnt/β-catenin signaling leading to inhibition of proliferation in pancreatic tumor cell lines. Additionally, we demonstrate for the first time that the combination of PG545 with gemcitabine has strong synergistic effects on viability, motility and apoptosis induction in several pancreatic cell lines. In an orthotopic xenograft mouse model, combination of PG545 with gemcitabine efficiently inhibited tumor growth and metastasis compared to single treatment alone. Also, PG545 treatment alone decreased the levels of β-catenin and its downstream targets, cyclin D1, MMP-7 and VEGF which is consistent with our *in vitro* data. Collectively, our findings suggest that PG545 exerts anti-tumor activity by disrupting Wnt/β-catenin signaling and combination with gemcitabine should be considered as a novel therapeutic strategy for pancreatic cancer treatment.

## INTRODUCTION

Pancreatic ductal adenocarcinoma (PDAC) the most common tumor of the pancreas, is an aggressive cancer that is highly proliferative, chemoresistant, angiogenic and antiapoptotic with a dismal 5 year survival rate of less than 5% [1, 2]. Monotherapy with gemcitabine, a deoxycytidine nucleoside analog which is currently used in the treatment of patients with PDAC, is not very effective in reducing the metastatic potential of these tumors [3] leading to the suggestion that a combination of gemcitabine with targeted agents may be more effective in combating metastasis of PDAC. Of all the signaling pathways altered in PDAC, the Wnt/β-catenin canonical pathway is considered to play a critical role in promoting angiogenesis, tumor progression, dysregulation of cell cycle and apoptosis, carcinogenesis [4-6] and metastasis in pancreatic cancer [7]. Wnt3a is considered a major initiating factor in the Wnt/β-catenin canonical pathway [8] and Wnt7a has been shown to be highly expressed in pancreatic cancer cell lines [9]. Thus, a novel therapy targeting Wnt signaling with combination of gemcitabine can be considered as a potent therapeutic strategy to improve the overall survival of patients with PDAC.

Heparanase, an endo-β-D-glucuronidase, is involved with tumor angiogenesis and metastasis via the regulation of heparan sulfate (HS) cleavage in several cancers [10, 11]. A new heparanase inhibitor/HS mimetic, PG545, suppressed tumor growth and metastasis in various solid tumors and metastasis models [12-14] and also dramatically increased the overall survival in a syngeneic 4T1 breast cancer model which was associated with down regulation of heparanase in primary tumor and lung metastases [15]. This compound also acts by inhibiting the binding of growth factors such as FGF-2 and VEGF to HS leading to reduced signaling through their cognate receptors [12]. Though all of these reports attest to the anti-angiogenic, anti-tumor and/or anti-metastatic activity of PG545, the focus of these studies tended to be just on the changes observed within the tumor microenvironment (including host cells) rather than on specific molecular switches or signaling pathways within the actual tumor cells. Thus, in the present study, the molecular mechanism of PG545 alone or its combination with gemcitabine was investigated in association with Wnt/β-catenin signaling in various pancreatic cells *in vitro* and *in vivo*.

## RESULTS

### PG545 suppresses Wnt/β-catenin signaling in pancreatic cancer cells

**Figure 1:**
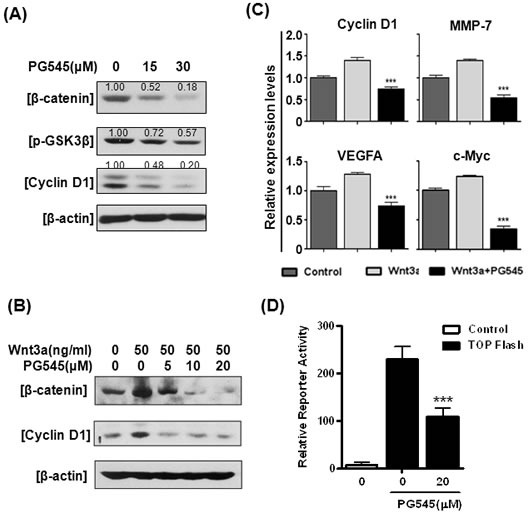
Effects of PG545 on Wnt/β-catenin signaling in pancreatic cancer cells (A) AsPC-1 cells were treated with various concentrations of PG545 for 6 h. (B) AsPC-1 cells were treated with various concentrations of PG545 for 6 h with or without 50 ng/ml of Wnt3a and subjected to western blotting. (C) AsPC-1 cells were incubated with PG545 (30 μM) and/or Wnt3a (100 ng/mL) for 24 h and the mRNA expressions of β-catenin downstream target genes were analyzed by Real-Time quantitative RT-PCR. (D) AsPC-1 cells containing β-catenin binding promoter fused with luciferase gene or control cells were incubated with PG545 for 24 h. ***P < 0.001 vs. TOPflash without PG545.

PG545 dramatically decreased the levels of β-catenin, phosphorylation of GSK3α/β and its downstream target, Cyclin D1 in all tested pancreatic cancer cells (AsPC-1, MiaPaCa-2, and Capan-1) in a concentration-dependent manner (Fig. 1A and S1A). In order to determine if the decreased β-catenin resulted from blockade of the canonical Wnt pathway, the effect of PG545 was assessed in the presence of the ligand Wnt3a. PG545 reduced the Wnt3a-induced increase of β-catenin and Cyclin D1 in AsPC-1 cells (Fig. 1B). Conversely the suppression of β-catenin and Cyclin D1 level by PG545 was rescued by Wnt3a at 50 ng/ml (Fig. S1B). We found that PG545 consistently attenuated the expression of Cyclin D1, MMP-7, VEGF and c-Myc by RT-qPCR (Fig. 1C) and the transcriptional activity of β-catenin by luciferase assay in AsPC-1 cells (Fig. 1D) [16]. In addition, PG545 also suppressed the proliferation of AsPC-1 and Panc-1 cells in a concentration-dependent manner (Fig. S1C).

### PG545 blocks Wnt binding to cell surface

**Figure 2:**
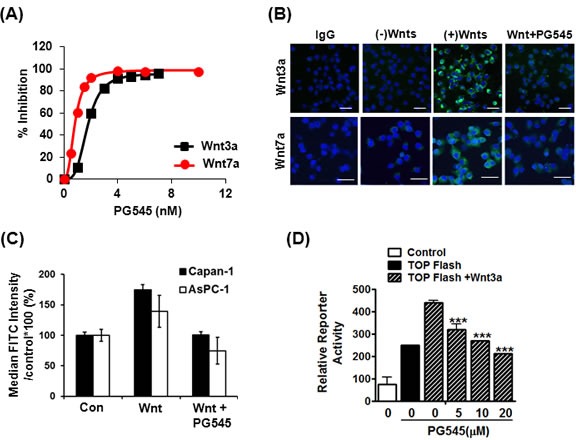
Disruption of HS-mediated Wnt/β-catenin signaling by PG545 (A) Inhibition of Wnt-heparin binding by PG545. Representative response curves for PG545 inhibition of Wnt3a and Wnt7a binding to immobilized heparin. Multiple response curves were used to determine IC_50_ values of 1.91 ± 0.09 nM for Wnt3a (n = 5) and 0.97 ± 0.12 nM for Wnt7a (n = 6). (B) Blocking effect of PG545 at the binding between recombinant Wnt and AsPC-1 cells was assessed by immunofluorescence. AsPC-1 cells were incubated with PG545 (20 μM) and/or recombinant His-tagged Wnt3a or Wnt7a (200 ng/mL) for 6 h as shown in MATERIALS AND METHODS. Scale bars; 5 μm. (C) Flow cytometric analysis for detection of FITC positive cells was used to observe the inhibition by PG545 of Wnt3a binding to Capan-1 and AsPC-1 cells. Cells were incubated with or without PG545 (20 μM) and/or recombinant His-tagged Wnt3a (100 ng/mL) for 2 h at 4°C. Y-axis represents the % of median FITC intensity indicating binding of Wnt ligands to cell surface. (D) Dose-dependent response for β-catenin transcriptional activation with increasing concentrations of PG545. AsPC-1 reporter and control cells were incubated with Wnt3a (100 ng/ml) and the indicated concentrations of PG545 for 24 h. ***P < 0.001 vs. TOPflash + Wnt3a without PG545.

To determine whether PG545 mediated down regulation of Wnt/β-catenin was dependent on proteosomal degradation, we treated the cells with 5μM proteasome inhibitor MG132. MG132 treatment rescued the down regulation of β-catenin by PG545 in Wnt3a treated AsPC-1 cells (Fig. S2A), indicating that proteasomal degradation, not transcriptional regulation, is responsible for modulating β-catenin levels. Since Wnt binds to the heparan sulfate moieties on HSPGs, we hypothesized that the highly sulfated HS mimetic PG545 would compete with HSPGs for Wnt [17, 18]. Therefore, we examined the interaction between Wnt3a or Wnt7a and PG545 using heparin-binding functional assays for these proteins. Here, PG545 potently inhibited the interaction of immobilized heparin to both Wnt proteins with IC_50_ values of 1.91 ± 0.09 nM for Wnt3a and 0.97 ± 0.12 nM for Wnt7a (Fig. 2A). Next, we evaluated the capacity of PG545 to inhibit the cell surface-binding ability of Wnts. Immunofluorescence analysis showed that the interaction between Wnt3a and Wnt7a and the surface of AsPC-1 cells was abrogated by PG545 (Fig. 2B). Furthermore, FACS analysis revealed that PG545 blocked the Wnt-cell surface interaction in AsPC-1 and Capan-1 cells (Fig. 2C). Consistently, the binding of Wnt7a at the cell surface was reduced in a concentration dependent fashion by PG545 (Fig. S2C). We confirmed that PG545 attenuated the β-catenin transcriptional activity induced by Wnt3a (Fig. 2D). Taken together, these data suggest that PG545 blocks the HSPG-dependent interaction of Wnt proteins to the cell surface by directly binding to the Wnt HS-binding site.

### PG545 promotes apoptosis in pancreatic cancer cells via inhibition of the Wnt/β-catenin signaling

**Figure 3:**
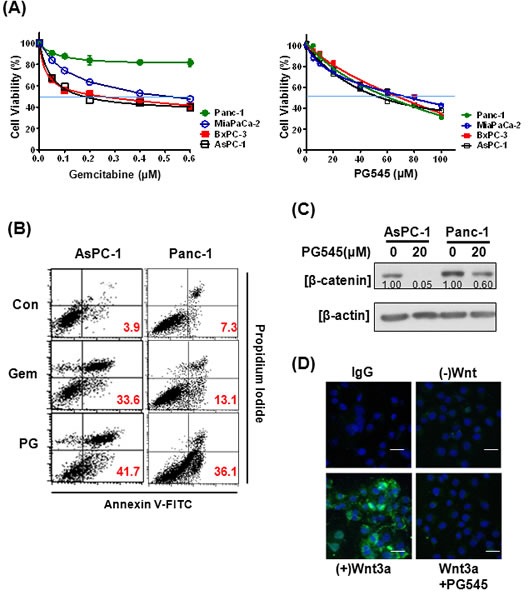
Effects of PG545 and gemcitabine treatment on pancreatic cancer cells (A) AsPC-1, BxPC-3, MiaPaCa-2, and Panc-1 cells were treated with various concentrations of gemcitabine (0.05 to 0.6 μM) for 72 h or PG545 (5 to 100 μM) for 24 h. Cell viability was measured by MTT assay. (B) AsPC-1 and Panc-1 cells were treated with PBS (control), gemcitabine (50 ng/ml) or PG545 (40 μM). After staining with Annexin V-FITC and PI, the apoptotic cells were analyzed by flow cytometer. The numbers in each plot indicate the percentage of apoptotic cells, i.e. positive staining for both Annexin V and PI. (C) The cells treated with PG545 (20 μM) for 6 h were subjected to Western blotting. Experimental conditions of (D) using Panc-1 cells were same in Figure 2B. Scale bars; 5 μm.

Due to the importance of gemcitabine in treatment of pancreatic cancer, we compared the potency of PG545 against four pancreatic cell lines and compared this to gemcitabine. Based on cell viability data, Panc-1 was considered less susceptible to gemcitabine compared with AsPC-1, MiaPaCa-2 and BxPC-3 (Fig. 3A). PG545 was effective against all four pancreatic cancer cells tested, including Panc-1, with IC50 values ranging from 51 to 70 μΜ. Similarly, flow cytometric analysis revealed that PG545 dramatically increased the Annexin V-PI double stained apoptotic population to 36.1% in Panc-1 cells compared to untreated control (7.3%), while gemcitabine-induced apoptosis only in AsPC-1, but not in Panc-1 or AsPC-1 cells previously cultured to become less susceptible to gemcitabine (termed gemcitabine resistant or GR cells) (Fig. 3B and Fig. S3). Next, we evaluated the anti-tumor effect of PG545 on Wnt/β-catenin signaling in Panc-1. PG545 reduced the levels of β-catenin in Panc-1, similar to the response seen in MiaPaCa-2 and Capan-1 cells (Fig. S1A), albeit to a lesser extent compared to AsPC-1 (Fig. 3C). Additionally, we found that PG545 completely blocked the binding of Wnt3a or Wnt7a to the surface of Panc-1 cells (Fig. 3D, S2B and Fig. S2D), similar to what was observed for AsPC-1 and Capan-1 cells (Fig. 2B and C). These data suggest that inhibiting the Wnt/β-catenin signaling pathway should be considered a new mechanism of action for PG545 in pancreatic cancer.

### PG545 exerts synergistic effects with gemcitabine in pancreatic tumor cells

**Figure 4:**
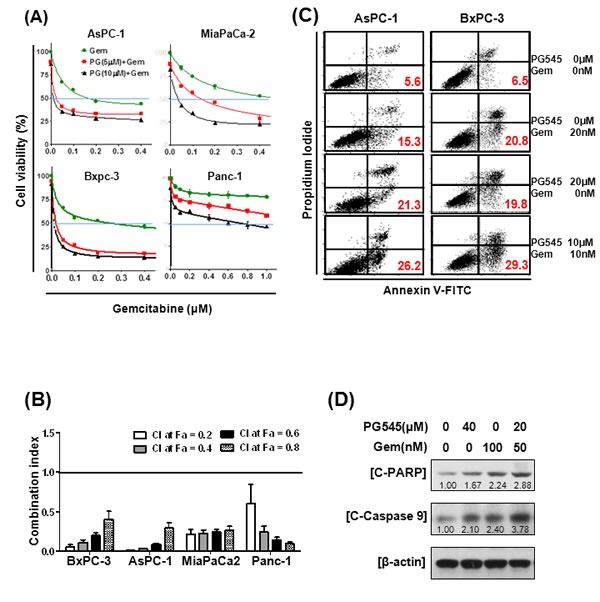
Synergistic effects of PG545 and gemcitabine co-treatment on pancreatic cancer cells (A) AsPC-1, BxPC-3, MiaPaCa-2, and Panc-1 cells were placed on 96-well plates and treated with gemcitabine (0.05~0.6 μM) alone or in combination with 5 or 10 μM of PG545. Cell viability was measured by MTT assay. Data represent means ± S.D. from three independent experiments. (B) Combination index (CI) values with fraction affected (Fa) between gemcitabine and PG545 in AsPC-1, BxPC-3, MiaPaCa-2 and Panc-1 cells was calculated using Calcusyn software. (C) and (D) AsPC-1 cells were treated with indicated concentrations of PG545 and gemcitabine for 48h. (C) The other procedures of apoptosis assay are same in Fig. 3B. (D) Cell lysates were subjected to Western blotting.

Because single agent gemcitabine treatment of pancreatic cancer has not greatly improved patient prognosis [3], considerable research has been conducted to find treatments that work more effectively in combination with this agent. We combined gemcitabine with PG545 to determine if, together, they provide synergistic benefits. The combination treatment of PG545 and gemcitabine showed greater potency than gemcitabine alone in reducing viability of all the pancreatic cancer cell lines, AsPC-1, MiaPaCa-2, BxPC-3 and Panc-1 cells (Fig. 4A). Significantly, the combination index (CI) values below 0.5 indicated that the combination treatment showed strong synergy in all four tested cells (Fig. 4B). CI values of <1 indicate synergistic, =1 additive and >1 antagonistic behavior. Evidence of a synergistic effect of PG545 treatment with gemcitabine was also shown in gemcitabine-resistant cells (Fig. S3 and S4), leading to dramatic decreases in the viability and enhanced apoptosis of these cells, compared with parental cells or gemcitabine alone (Fig. S3 and 4A). Given CI values were under 1 across most fractions affected (Fa) (Fig. S4B), systematic assessment of the potential for PG545 to overcome gemcitabine resistance *in vivo* is warranted but considered beyond the scope of the current studies.

Wound healing assays demonstrated that combination treatment either with half the dose or the same concentration used for single treatment was more effective in inhibiting the motility of AsPC-1 cells than PG545 or gemcitabine alone (Fig. S5). Furthermore, flow cytometric analysis showed combination treatment with half the concentrations of the drugs used for single treatment increased the Annexin V-PI double stained apoptotic population compared to PG545 or gemcitabine alone (Fig. 4C). Consistently, immunoblot analysis showed combination treatment enhanced the levels of the pro-apoptotic markers, cleaved PARP and activated Caspase-9 compared to single treatment alone (Fig. 4D). Conversely, PG545 showed no activation of the alternative apoptotic Caspase 8 pathway, although gemcitabine alone did elicit a response (Fig. S6).

### PG545 and gemcitabine combination suppresses tumor growth and metastasis in orthotopic model

**Figure 5:**
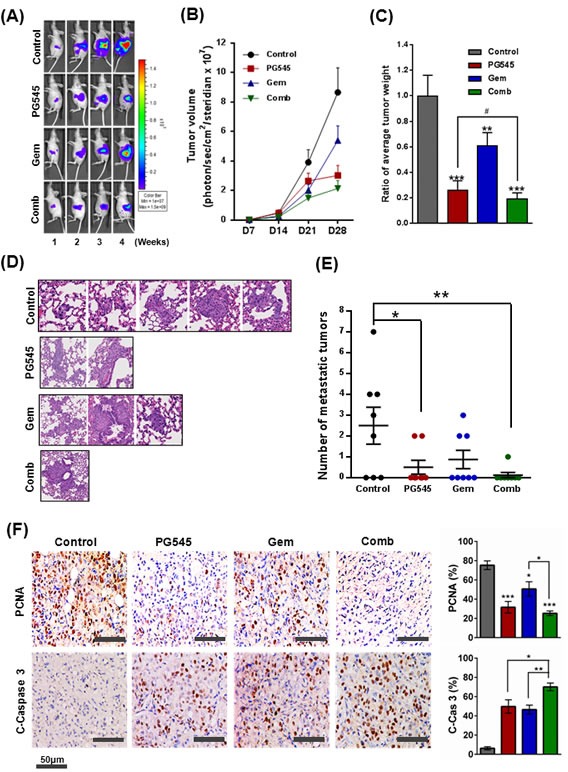
Inhibition of tumor growth and metastasis by PG545 in AsPC-1 orthotopic xenograft mouse model Treatment groups: Control group data, grey or black symbols and bars; PG545 group data, red; Gem group data, blue; Combination group (at half the PG545 and Gem doses) data, green. See Materials and Methods for experimental details. (A) Bioluminescence IVIS images of orthotopically implanted pancreatic tumors in live and anesthetized mice (n=7-8). (B) Tumor volume during the experiment. (C) Final tumor weight at termination of experiment. Tumor data expressed relative to average tumor weight of control group. **P < 0.01, ***P < 0.001 vs. control; #P < 0.025 vs. PG545 alone. (D) Lung tissue sections were stained with Hematoxylin and Eosin. (E) Number of lung metastases. *P < 0.05, **P < 0.01. (F) Representative examples of immunohistochemical staining for PCNA and Cleaved Caspase-3 in tumor sections with histograms showing quantitation of staining.

The effect of PG545-gemcitabine combination on primary tumor growth and metastasis was evaluated in orthotopic AsPC-1 bearing nude mice. Tumor growth of four groups was measured once a week for four weeks using *in vivo* image analyzers as described in the methods section (Fig. S7). Tumor volume in mice treated with combination of PG545 and gemcitabine was reduced by 4-fold compared to the vehicle injected group and was significantly less than single agent, gemcitabine at day 28 (Fig. 5A and B). At necropsy, the measured tumor weight and size of mice in each group showed that the combination treatment was more effective at reducing cancer progression compared to all of the other groups (Fig. 5C and Fig. S8B). To further evaluate tumor progression, we counted the number of metastatic lesions in the lung. The combination group showed a dramatically decreased number of lung metastasis compared to PG545 or gemcitabine alone treated group (Fig. 5D, E and Fig. S8C). Consistent with these morphological data, immunohistochemistry revealed the decreased expression of the cell proliferation marker PCNA and increased levels of apoptotic marker cleaved-Caspase 3 in the combination treatment group compared to single treatment groups and control (Fig. 5F). These data demonstrate that PG545-gemcitabine combination treatment leads to significantly enhanced antitumor activity in a mouse model of PDAC. There was no significant body weight loss in PG545 or combination treated group compared to control group (Fig. S8A).

### PG545 suppresses the Wnt/β-catenin signaling in pancreatic tumor xenografts

**Figure 6:**
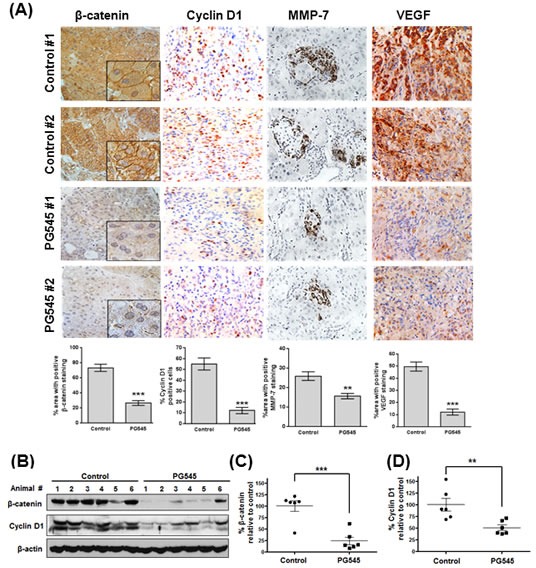
PG545 inhibits β-catenin signaling in AsPC-1 orthotopic xenograft mouse model (A) Immunohistochemical analysis of β-catenin and β-catenin-regulated proteins, Cyclin D1, MMP-7 and VEGF in pancreatic tumor tissues from mice. Quantitation of staining was performed using 10 fields per analyte. **P < 0.01, ***P < 0.001. (B) Frozen tumor tissues were homogenized on ice and the extracts were subjected to Western blotting. (C) β-catenin and (D) Cyclin D1 levels were quantified by Image J software and plotted relative to the control group in Fig. 6B. **P < 0.01, ***P < 0.001 vs. control.

Tumor samples from the xenograft study presented in Figure 5 were further analyzed to examine the biochemical mechanism underlying the anti-tumor activity of PG545. IHC analysis of xenografts showed that the expression of VEGF, MMP-7, Cyclin D1 and β-catenin was significantly decreased in PG545 treated group compared to untreated control (Fig. 6A). In contrast, staining for VEGFR2, a major receptor involved in mediating VEGF signaling, showed no differences across the 4 treatment groups (Fig. S9). Consistent with the IHC data, immunoblotting showed that β-catenin and cyclin D1 levels were decreased by70% and 45%, respectively in the PG545 treated group compared to the untreated control group (Fig. 6B, C and D), which is also consistent with our *in vitro* immunoblotting and RT-qPCR results (Fig. 1).

## DISCUSSION

Pancreatic ductal adenocarcinoma (PDAC) occurs, in the majority of cases, with early locoregional spread and distant metastasis at diagnosis, leading to dismal prognosis and limited treatment options. Standard chemotherapy, typically with gemcitabine, provides only modest benefit to patients [19] and thus novel pathways for intervention are being investigated. The Wnt ligands are secreted glycoproteins that have critical roles in a variety of cellular processes, including survival, differentiation, migration and proliferation through regulation of β-catenin stability [16, 20]. Recently, aberrant levels of β-catenin have been detected in pancreatic cancer [21] and it is now apparent that ligand-mediated activation of the Wnt/β-catenin pathway is required for the initiation of pancreatic cancer and Wnt signaling is critical for progression of the disease [6].

As the enzyme primarily responsible for the degradation of HS chains, heparanase has been shown to modify tumor cell responses to HS-binding of Wnt3a such as effects on cell proliferation and adhesion, in addition to its HS-chain degrading activity [22]. Given the high affinity binding of Wnt ligands to HS [23] and the link between altered HSPGs, heparanase, and tumor cell metastasis [24-26], investigating HS mimetics/heparanase inhibitors effect on Wnt signaling was considered a novel approach to discover new therapeutic options for pancreatic carcinomas. A HS mimetic M402 currently in Phase I/II trials in pancreatic cancer was designed to inhibit tumor-host interactions including VEGF, FGF2, SDF-1 and heparanase [27]. However, to the best of our knowledge, the ability of HS mimetics/heparanase inhibitors to bind and modulate the Wnt signaling pathway has not been investigated. Here, we identify a new role for PG545 in the blockade of Wnt signaling and demonstrate for the first time that a HS mimetic can repress this significant tumor-promoting pathway. PG545 interacts with Wnt3a and Wnt7a to preventing them from binding to their receptors, LRP5/6 and Frizzled (Fig. 2), resulting in significantly decreased β-catenin levels (Fig. 1), suppressed tumor growth and metastasis in pancreatic tumors (Fig. 5).

A recent study by Ostapoff and colleagues demonstrated that PG545 suppresses the proliferation, migration and colony formation of pancreatic ductal adenocarcinoma (PDAC) cells [14]. Moreover, the same group showed PG545 treatment of a *mPDAC* genetic model also led to a change in the localization of β-catenin within tumor cells - in the untreated controls, β-catenin was associated with the nucleus but in the PG545 treated tumors, it was associated with the membrane and in the cytoplasm. This is broadly consistent with the notion that PG545 blocks Wnt/β-catenin signaling, thus preventing β-catenin from entering the nucleus in significant quantities. In the present study, we show equally profound, but slightly divergent, effects on the levels and localization of β-catenin within AsPC-1 xenograft tumor cells treated with PG545 (Fig. 6). The untreated controls show higher β-catenin levels throughout the cells compared to the PG545 treated tumors indicating that inhibition of Wnt-receptor binding led to a systemic reduction in the amount of β-catenin. The results of PG545 inhibition of this systemic suppression of β-catenin can be seen in the reduced levels of proteins whose expression is driven by β-catenin including Cyclin D1, MMP-7 and VEGF (Fig. 6) and in the induction of apoptosis (Fig. 3 and 4). The induction of cleavage of the apoptotic proteins caspase 3, caspase 9 and PARP has been linked to blockade of Wnt/β-catenin signaling and provides further evidence that promotion of apoptosis by PG545 was being driven by Wnt inhibition [28-31]. The contrasting effects of PG545 on β-catenin in our AsPC-1 xenograft model compared to the *mPDAC* genetic model described by Ostapoff and colleagues may suggest differences in β-catenin regulation between human and mouse pancreatic tumors.

Given the low survival rates in PDAC, significant efforts are underway to seek agents to combine with gemcitabine. Based on our data, PG545 combined with gemcitabine led to a synergistic inhibitory effect on cancer cell survival and motility (Fig. S5), induction of apoptosis (Fig. 3B and 4C and D), enhanced antitumor activity (Fig. 5A, B, C) and anti-metastatic activity (Fig. 5E) versus either agent alone and could possibly overcome gemcitabine resistance as evidenced by *in vitro* data (Fig. 4A, B, S3 and S4). However, the potential utility of this combination in the clinic would firstly focus on improving the overall response rate/blockade of metastatic spread in those patients receiving gemcitabine as standard-of-care. Patients who subsequently become resistant to gemcitabine would have treatment withdrawn to facilitate second-line treatment options. Therefore, consistent with the data herein the objective of an initial clinical investigation of PG545 and gemcitabine would be to enhance the overall response in patients most likely to respond to gemcitabine.

PG545 is a clinically relevant HS mimetic (clinicaltrials.gov identifier NCT02042781) which, in addition to possessing anti-angiogenic properties, also acts as a heparanase inhibitor which may differentiate its mechanism(s) of action from approved angiogenesis inhibitors [13, 15]. Herein, we report that further to the previously published findings on PG545′s ability to interfere with angiogenic growth factors, this compound also inhibits Wnt signaling within tumor cells which leads to reduced levels of β-catenin, VEGF, MMP-7 and Cyclin D1 (Fig. 6). Based on these data, the inhibition of Wnt signaling by PG545 represents a novel effect on the tumor rather than the host cell, and thus may explain the synergistic effects on proliferation, motility and apoptosis in pancreatic cancer cell lines *in vitro* and an enhanced antitumor response *in vivo*, when administered in combination with gemcitabine. An alternative, or perhaps complementary, explanation for the observed synergy is that PG545 and gemcitabine target different pro-tumor pathways, thus eliminating the redundancy that allows tumor cells to adapt to treatment and continue to proliferate and spread. These findings highlight the need to better understand the recently described interaction between Wnt signaling and heparanase in cancer biology [22].

In summary, we clearly demonstrate that PG545 directly binds with Wnts leading to a blockade of ligand-receptor binding (Fig. S10). This inhibitory effect on β-catenin stability resulted in suppression of tumor growth and metastasis and induction of apoptosis in PDACs. Furthermore, co-treatment of PG545 and gemcitabine showed the synergistic antitumor activity in PDACs compared to single agent treatment. Overall, these findings suggest that combination of PG545 and gemcitabine can be a potent therapeutic strategy for pancreatic cancer treatment.

## MATERIALS AND METHODS

### Cell cultures and reagents

Pancreatic cancer cell lines, AsPC-1, MiaPaCa-2, Panc-1, BxPC-3 and Capan-1 were obtained from the American Type Culture Collection (ATCC, USA). AsPC-1, BxPC-3 and Capan-1 cells were cultured in RPMI 1640 medium supplemented with 10% FBS and 1% antibiotic (Welgene, South Korea), while MiaPaCa-2 and Panc-1 cells were cultured in DMEM with 10% FBS and 1%. Cancer cell lines were authenticated by short tandem repeat (STR) DNA profiling analysis. Recombinant human Wnt3a and Wnt7a proteins were purchased from R&D Systems (USA). PG545 was provided by Progen Pharmaceuticals (Australia) and gemcitabine was from Sigma Chemical (USA). The recombinant His-tagged Wnt3a and Wnt7a proteins were from Proteintech Group (USA).

### Cytotoxicity assay

To evaluate the synergistic effect of PG545 and gemcitabine on cell viability, 3-(4,5-dimethylthiazol-2-yl)-2,5-diphenyltetrazolium bromide (MTT) assay was performed. Briefly, four pancreatic cancer cells such as AsPC-1, MiaPaCa-2, Panc-1 and BxPC-3 cells (1×10^4^ cells/well) were seeded onto 96-well culture plate and exposed to various concentrations of PG545 and/or gemcitabine. The viability of cells was analyzed as previously described [32].

### Luciferase assay

Transfection was carried out with X-tremeGENE HP DNA transfection Reagent (Roche Applied Sciences, USA) according to the manufacturer's instructions. Luciferase assays were performed using the Dual luciferase assay kit (Promega, USA). The TOPflash reporter plasmid kindly gifted by Dr. Sangtaek Oh (Kookmin University, Korea).

### Proliferation assay

To examine the anti-proliferative effect of PG545, Panc-1, and Capan-1 cells (1×10^4^ cells/well) were seeded onto 96-well cell culture plate and exposed to various concentrations of PG545 for 24 h. Proliferation assays were performed using a BrdU Cell Proliferation assay kit (Roche Applied Sciences, USA) according to the manufacturer's protocol.

### Western blotting

Cells were lysed in RIPA buffer (50mM Tris-HCl, 150mM NaCl, 2mM EDTA, and 1% TritonX-100) containing protease inhibitors (Roche, Germany), and phosphatase inhibitors (Sigma, USA). The protein samples were separated on 8 to 15% SDS-polyacrylamide gels, and transferred to nitrocellulose membranes. Membranes were incubated with primary antibodies diluted in 3% BSA in PBS-Tween20 (1:500-1:2000) overnight at 4°C, washed three times with PBS-Tween20, and incubated with HRP-conjugated secondary antibodies. Expression was visualized by using ECL Western blotting detection reagent (GE Healthcare, UK)

### Apoptosis detection by Annexin V-PI double staining

Apoptosis of the PG545 and/or gemcitabine-treated cells was determined as previously described [32]. Cells were quantitated by double staining with Annexin V-FITC and propidium iodide (PI) using the Annexin V-Apoptosis Detection kit (Biovision, USA). Apoptotic cells were analyzed by FACSCalibur (Becton Dickinson, USA) to define as those positive cells for Annexin V with or without PI staining.

### Real-Time Quantitative PCR (RT-qPCR)

Total RNA was isolated from AsPC-1 cells with QIAzol (Invitrogen, USA). A reverse transcription kit (Promega, USA) was used to construct the template cDNA. RT-qPCR was performed with the LightCycler480 instrument (Roche Applied Sciences, USA). The mRNA level of GAPDH was used to normalize the expression of genes of interest. The primers used are in Table S1.

### Wnt heparin binding assay

The binding of Wnt proteins to heparin was assayed in heparin-coated 96-well microplates using antibody detection. All steps were performed at 22°C. Heparin was immobilized onto DNA-Bind microplates (Corning, USA) via an amine group introduced at the reducing end of the heparin polymer (Supplementary Methods).

### Immunofluorescence assay

Cells treated with recombinant human His-Wnt3a or Wnt7a protein in the presence or absence of PG545 were fixed with 4% paraformaldehyde, blocked in 0.1% Triton X-100, 5% BSA in PBS for 30min at 4°C and incubated with FITC-conjugated anti-His tag (Ab Chem, USA) overnight at 4°C. Immunostained cells were mounted with mounting medium containing DAPI (1.5 μg/mL) (Vectashield, USA) and visualized by using Olympus FLUOVIEW FV10i confocal microscope.

### Wnt binding assay by FACS

Cells were treated with recombinant human His-Wnt (100 ng/ml) with or without 10 μΜ PG545 at 4°C. His-Wnt treated cells were harvested and washed with cold PBS thrice, and then incubated with FITC-conjugated anti-His tag (Ab Chem, USA) for 1 h at 4°C. The antibody-labeled cells were fixed with 4% paraformaldehyde/5% BSA in PBS and the immunolabeled cells were analyzed by FACSCalibur (Becton Dickinson, USA).

### Synergy between PG545 and gemcitabine by combination index

To determine the synergy of PG545 and gemcitabine, cytotoxicity assay was performed in the pancreatic cancer cell lines. Following the determination of IC_50_ for each drug, synergy between PG545 and gemcitabine was evaluated by the method of Chou Talalay [33] using CalcuSyn software (Biosoft, USA) to calculate combination index values.

### Animal groups and tumor monitoring using animal imaging system

Female nude mice between the ages of 5 weeks were obtained from the National Cancer Institute (USA), Harlan Laboratories (USA). This study was approved by and conducted in accordance with the policies set forth Institutional Animal Care and Use Committee Mayo clinic. One week after 1 × 10^6^ Luc-transfected AsPC-1 cells were injected orthotopically into the pancreas, the mice were randomized into the following four groups (n = 8) based on the bioluminescence measured after the first IVIS imaging: (Group 1, Control) untreated control (PBS, 100 μL), twice-weekly by i.p. injection; (Group 2, PG545) PG545 (20 mg/kg), twice-weekly by i.p. injection; (Group 3, Gem) gemcitabine alone (25 mg/kg), twice weekly by i.p. injection; and (Group 4, Combination) combination of PG545 (10 mg/kg) and gemcitabine (12.5 mg/kg), twice weekly by i.p. injection (see Supplementary Methods for measurement of tumor volumes). The combination treatment employed reduced doses of PG545 and gemcitabine to reduce the possibility of toxicity arising when PG545 was paired with this potent cytotoxic agent.

### Histopathological analysis

Immunohistochemistry was performed in tumor sections using the indirect avidin biotin-enhanced horseradish-peroxidase method. Antigen retrieval was performed after deparaffinization and dehydration of the tissue sections by microwave for 10 min in 10 mM citrate buffer (see Supplementary Methods for tumor sections). Quantitation of staining was performed by analyzing 10 images per tumor at 20 × magnification using LAS image analysis software (Leica). The quantification of stained cells or area in each image is relative to the total tissue image area and is presented as a percentage = [Number of positive cells or area (pixel)/total cell number or area (pixel)] × 100.

To count lung metastases, lung tissue sections were stained with hematoxylin and eosin (H&E). Counting metastatic lesions of lung tissue sections was performed by an investigator blinded to the treatment status of the sample.

### Statistical analyses

All data were presented as means ± standard deviation (SD). The statistically significant differences between control and PG545/gemcitabine treated groups were calculated by Student's t-test. A one-way ANOVA was used for comparison of multiple groups. Statistical difference was set at p values of <0.05 between control and PG545/gemcitabine treated groups. All experiments were carried out at least thrice.

## SUPPLEMENTARY MATERIAL FIGURES AND TABLE


