# Correction: The heparan sulfate mimetic PG545 interferes with Wnt/β-catenin signaling and significantly suppresses pancreatic tumorigenesis alone and in combination with gemcitabine

**DOI:** 10.18632/oncotarget.27216

**Published:** 2019-09-24

**Authors:** Deok-Beom Jung, Miyong Yun, Eun-Ok Kim, Jaekwang Kim, Bonglee Kim, Ji Hoon Jung, Enfeng Wang, Debabrata Mukopadhyay, Edward Hammond, Keith Dredge, Viji Shridhar, Sung-Hoon Kim

**Affiliations:** ^1^ College of Korean Medicine, Kyung Hee University, Seoul, South Korea; ^2^ Korean Medicine Clinical Trial Center, Kyung Hee University, Seoul, South Korea; ^3^ Department of Biochemistry and Molecular Biology, MN, USA; ^4^ Progen Pharmaceuticals Ltd, Brisbane, Queensland, Australia; ^5^ Experimental Pathology, Mayo Clinic College of Medicine, Rochester, MN, USA; ^*^ These authors contributed equally to this work; ^**^ These authors are senior authors

**This article has been corrected:** The IHC pictures of ‘Control #1 group’ and ‘PG545 #2 group’ have been removed from Figure 6. In addition, the ‘Cyclin D1 in Control 1 group’ is mistakenly similar to the IHC picture of ‘PCNA in Gem group’ already presented in Figure 5F. The corrected Figure 6 is shown below. The authors declare that these corrections do not change the results or conclusions of this paper.

Original article: Oncotarget. 2015; 6:4992-5004. 4992-5004. https://doi.org/10.18632/oncotarget.3214

**Figure 6:**
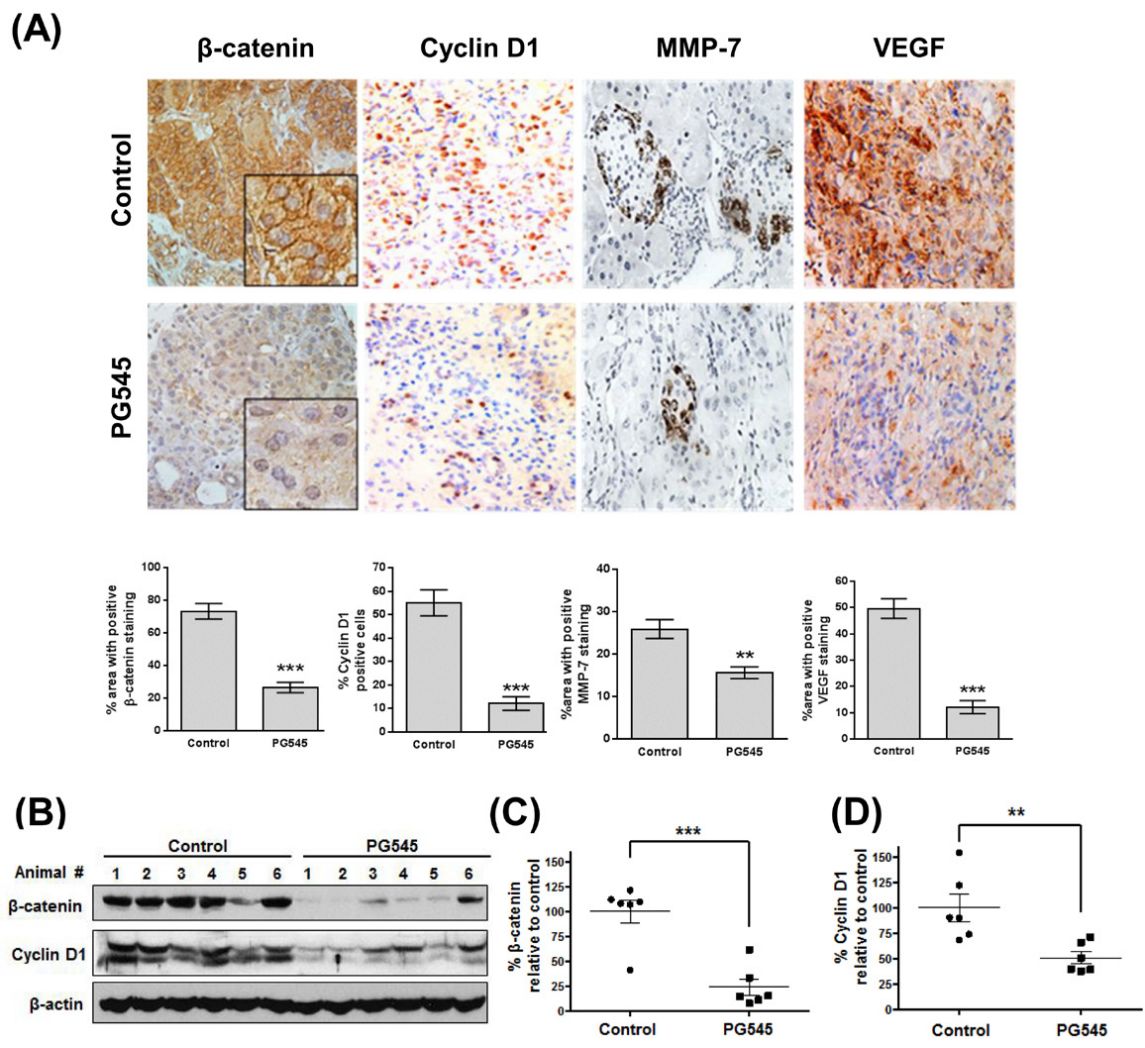
PG545 inhibits β-catenin signaling in AsPC-1 orthotopic xenograft mouse model. **(A)** Immunohistochemical analysis of β-catenin and β-catenin-regulated proteins, Cyclin D1, MMP-7 and VEGF in pancreatic tumor tissues from mice. Quantitation of staining was performed using 10 fields per analyte. **P & 0.01, ***P & 0.001. **(B)** Frozen tumor tissues were homogenized on ice and the extracts were subjected to Western blotting. **(C)** β-catenin and **(D)** Cyclin D1 levels were quantified by Image J software and plotted relative to the control group in Fig. 6B. **P & 0.01, ***P & 0.001 vs. control.

