# Supplementary material for: The heparan sulfate mimetic PG545 interferes with Wnt/β-catenin signaling and significantly suppresses pancreatic tumorigenesis alone and in combination with gemcitabine
Source: Oncotarget. 2014 Dec 31;6(7):4992–5004. doi: 10.18632/oncotarget.3214 (PMC4467129; doi:10.18632/oncotarget.3214)
Supplement: Supplementary file 1 [file oncotarget-06-4992-s001.pdf]

The heparan sulfate mimetic PG545 interferes with Wnt/β-catenin signaling and significantly suppresses pancreatic tumorigenesis alone and in combination with gemcitabine

Supplementary Material

Methods

Reagents

Caspase 8 antibody was purchased from Cell Signaling Technology (USA) and VEGFR2 antibody was purchased from Santa Cruz Biotechnology (USA).

Plate preparation for Wnt heparin binding functional assay

Varying concentrations of PG545 were equilibrated in buffer containing 20 mM Tris-HCl pH 7.4, 0.05% Tween 20, with 0.2 nM Wnt3a or 0.05 nM Wnt7a (R & D Systems, USA). After equilibration for 10 min, 100 µL aliquots were transferred to wells of the heparin-coated microplates sealed with adhesive film, and shaken for 2 hrs. The plates were blocked with BSA before use in assays. The plates were then washed three times with PBST before probing with primary antibodies (Abcam, UK, Wnt3a antibody was biotinylated) at 0.5 µg/mL in PBST for 1 hr. The plates were washed with PBST again and either streptavidin-HRP (Wnt3a assays, R & D Systems, USA) or goat anti-rabbit IgG conjugated to HRP (Wnt7a assays, Thermo, USA) added for 1 hr. Following final washing with PBST and then water, the plates were developed with 100 µL Ultra TMB peroxidase substrate (Thermo, USA) for 10 min before the reactions were stopped by the addition of 100 µL of 1 M H_2SO_4 and the absorbance at 450 nm measured (reference 620 nm).

Wound healing assay

Cells (1×10^5 cells/well) were seeded into 6-well tissue culture plate. After 24 hours, the center of the well was scratched with a new 1ml pipette tip, and gently washed twice with medium to remove the detached cells. The well was replenished with fresh medium containing PG545 and gemcitabine. The cells were grown for additional 48 hours and washed twice with 1 × PBS, then fixed and stained with Diff-Quick staining solution (Sysmex, Japan). The stained cells photographed with a camera mounted on a microscope (AXIO observer A1, Zeiss, Germany). The wound healing area was measured with the ImageJ, image processing program.

Establishment of gemcitabine resistant pancreatic cancer cell lines

Gemcitabine resistant AsPC-1 and BxPC-3 cells (AsPC-1/GR and BxPC-3/GR) were derived from each original parental cell lines by continuous exposure to gemcitabine (Sigma-Aldrich, USA) during several months in increasing concentrations up to 100 nM. The resistant cells were continuously maintained in a medium containing 50 nM of gemcitabine prior to each experiment.

Animal groups and tumor monitoring using animal imaging system

Tumor volumes were measured weekly by the bioluminescence IVIS Imaging System 200 using a cryogenically cooled imaging system coupled to a data acquisition computer running Living Image software (Xenogen Corp., Alameda, CA). Before imaging, animals were anesthetized in an acrylic chamber with 2.5% isoflurane/air mixture and injected i.p. with 40 mg/mL d-luciferin potassium salt in PBS at a dose of 150 mg/kg body weight. After 10 min of incubation with luciferin, mice were placed in a right lateral decubitus position and a digital grayscale animal image was acquired followed by acquisition and overlay of a pseudo color image. Signal intensity was quantified as the sum of all detected photons within the region of interest per second. Mice were imaged on days 0, 7, 14, 21, 24, and 31 of treatment. Treatment was continued for 4 weeks and all mice were sacrificed 1 week later. Primary tumors in the pancreas were excised and the final tumor volume (V) was measured as $V = 2 / 3\pi r^3$, where r is the mean of the three dimensions (length, width, and depth). Half of the tumor tissue was formalin fixed and paraffin embedded for immunohistochemistry and routine H&E staining. The other half was snap frozen in liquid nitrogen and stored at −80°C for immunoblotting.

Tumor sections for Immunohistochemistry

Tumor sections were cooled on bench top for 30 min, treated with 3% hydrogen peroxide in methanol for 10 min, and blocked with 6% horse serum for 30min at room temperature. Sections were then incubated with the primary antibody at 4°C overnight in humidity chamber. Sections were washed in PBST and incubated with secondary antibody (biotinylated goat anti-rabbit (1:150, Vector Laboratories, Burlingame, CA, USA) or biotinylated rabbit anti-rat IgG (1:150, Abcam, Boston, MA, USA) for 40min in humidity chamber. After further washes, the antibodies were detected with the Vector ABC complex/horseradish peroxidase (HRP) kit (Vector Laboratories, Burlingame, CA, USA), and color developed with 3,3′-diaminobenzidine tetrahydrochloride. For semiquantitation, ten photomicrographs (200×) were taken with a CCD camera, avoiding gross necrotic areas.

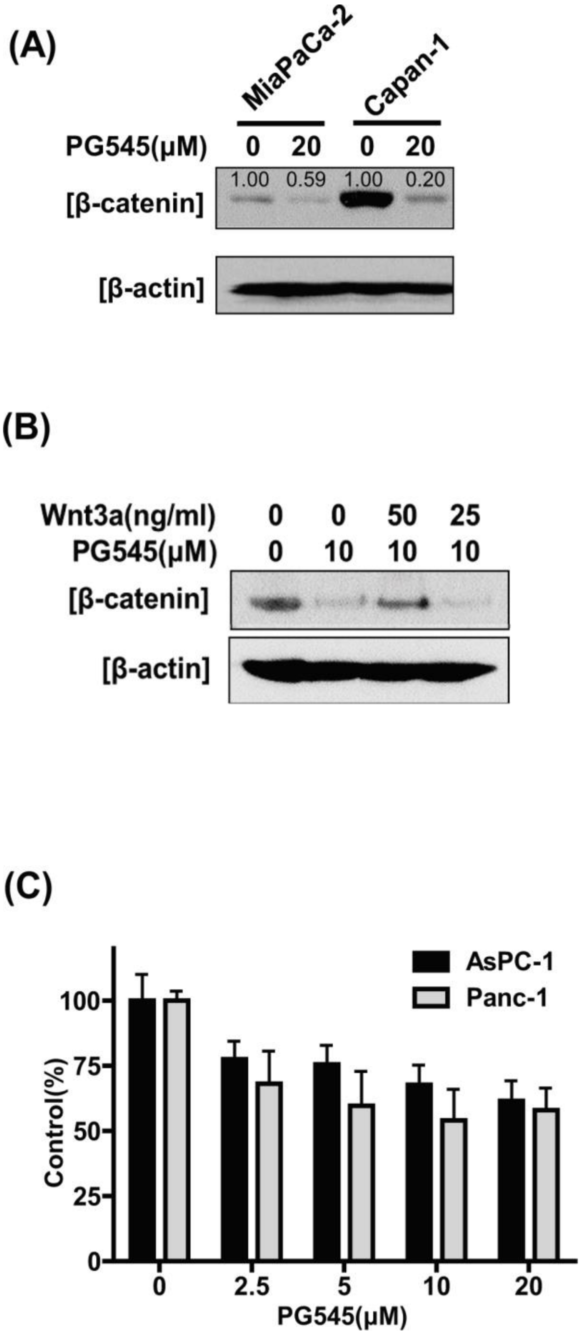

Figure S1: Effects of PG545 on Wnt/β-catenin signaling in pancreatic cancer cells.

(A) Various pancreatic cancer cells were treated with PG545 (20 μM) for 6h. (B) AsPC-1 cells were treated with different concentration of Wnt3a (25, 50 ng/ml) for 6 h in the presence of PG545 (10 ng/ml). β-catenin expression were determined by Western blotting. (C) The inhibition of AsPC-1 and Panc-1 cell proliferation by PG545 was measured using the BrdU assay after 24 h exposed to PG545 and Wnt3a (50 ng/ml).

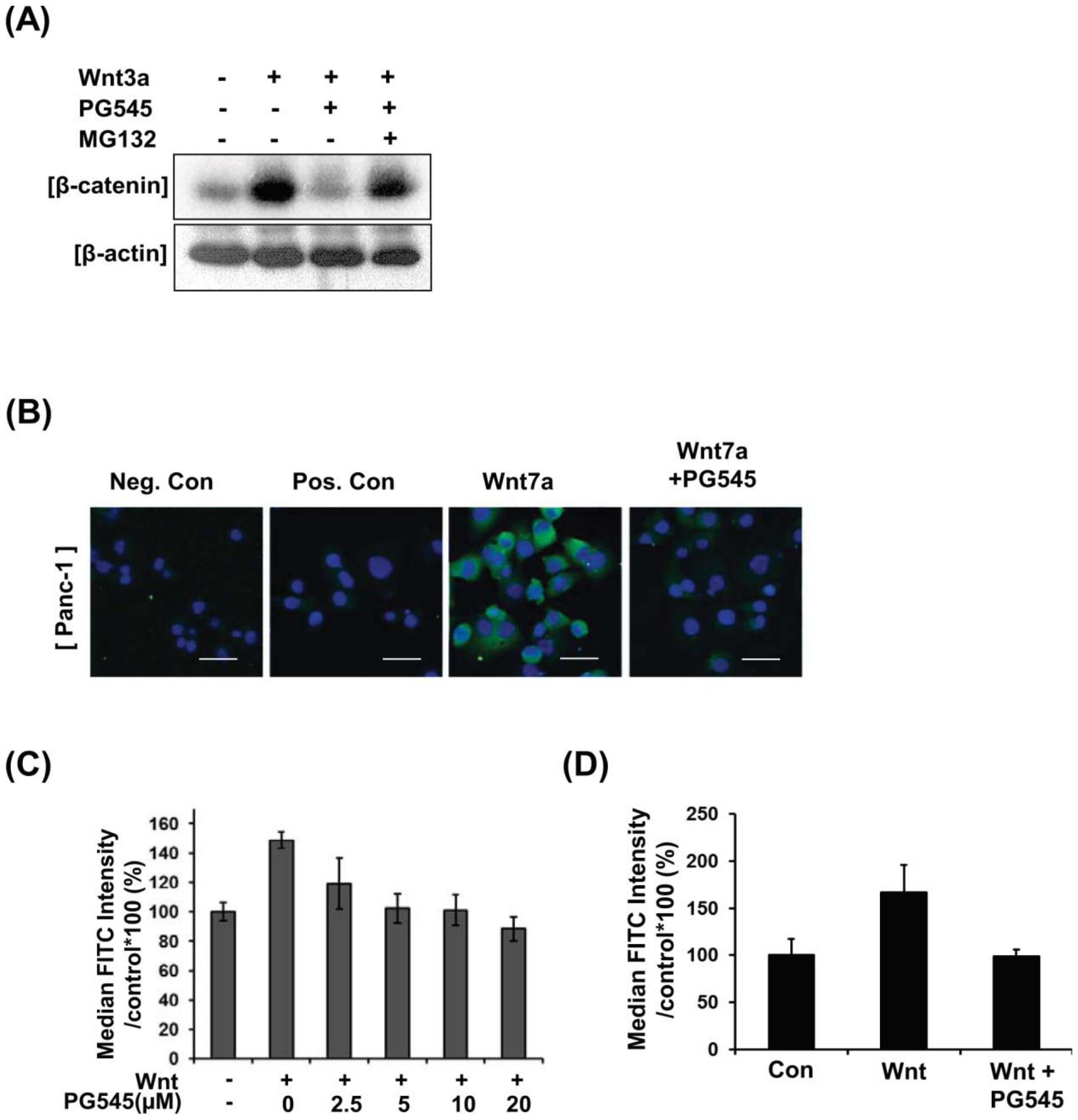

Figure S2: PG545 blocks Wnt7a binding to cells.

(A) AsPC-1 cells were incubated with or withoutWnt3a (50 ng/ml), PG545 (20 µM) and the proteasome inhibitor MG132 (5 µM) for 6 h. Cell lysates were subjected to Western blotting. (B) Blocking effect of PG545 on the binding of recombinant Wnt7a to Panc-1 cells as assessed by immunofluorescence. Panc-1 cells were incubated with PG545 (20 µM) and/or recombinant His-tagged Wnt7a (200 ng/mL) for 6 h. Staining procedures were described in Materials and Methods. Scale bars, 5 µm. (C) AsPC-1 cells were incubated with indicated concentrations of PG545 with or without recombinant His-tagged Wnt7a (100 ng/mL) for 2 h at 4°C and then flow cytometric analysis was conducted. (D) Flow cytometric analysis for detection of FITC positive cells was used to observe the inhibition by PG545 of Wnt7a binding to Panc-1 cells. Cells were incubated with or without PG545 (20 µM) and/or recombinant His-tagged Wnt3a (100 ng/mL) for 2 h at 4°C. Y-axis represents the % of median FITC intensity indicating binding of Wnt ligands to cell surface.

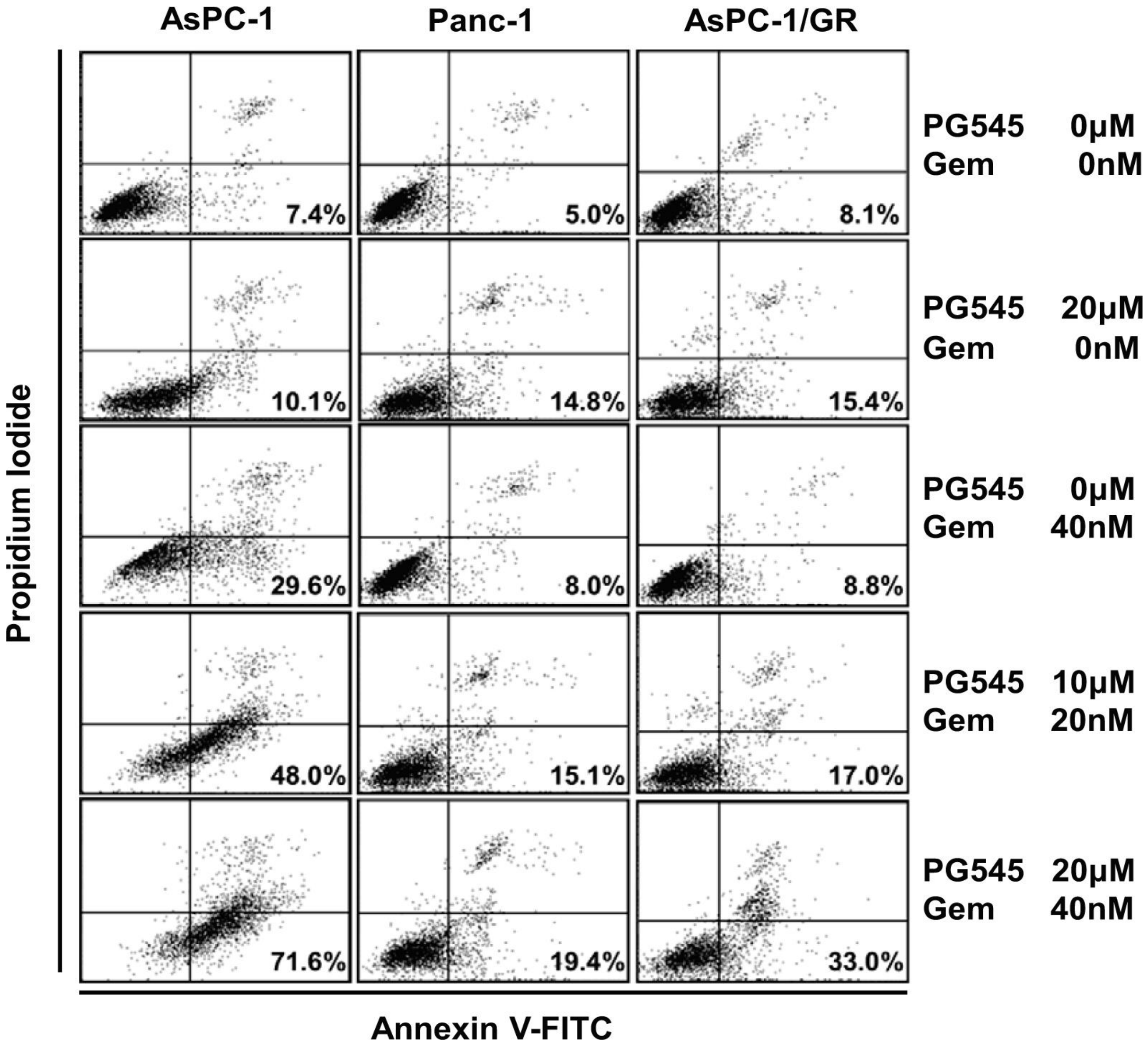

Figure S3: Combination effect of PG545 and gemcitabine on apoptosis induction in gemcitabine resistant pancreatic cancer cells.

AsPC-1, Panc-1 and AsPC-1/GR cells were treated with indicated concentrations of PG545 and gemcitabine for 48 hr. After staining the cells with annexin V-FITC and PI, the apoptotic cells were analyzed by a dot-plot using a flow cytometer. The numbers in each plot indicate the percentage of apoptotic cells, i.e. those staining positively for annexin V alone and both annexin V and PI. Gem: gemcitabine.

(A)

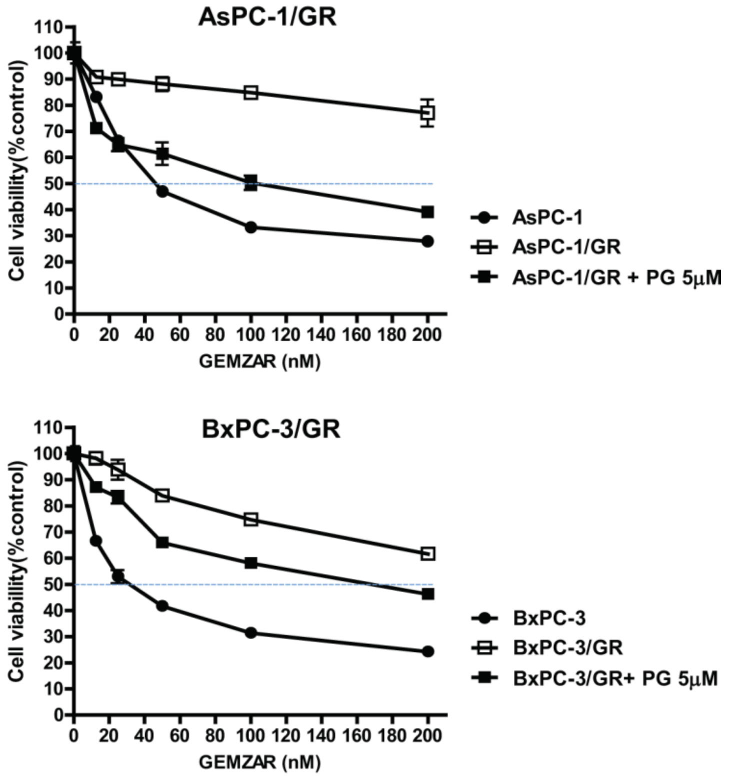

(B)

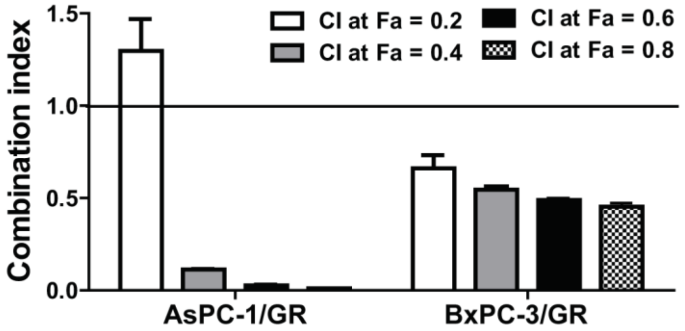

Figure S4: Synergistic effect of PG545 and gemcitabine co-treatment on gemcitabine resistant cells.

(A) AsPC-1/GR and BxPC-3/GR cells were plated on 96-well plates and treated with gemcitabine up to 200 nM, either alone or in combination with 5 μM of PG545 for 72 h. Cell viability was measured by MTT assay. Data represent means ± S.D. from three independent experiments. (B) Combination index (CI) values with respect to fraction affected (Fa) between gemcitabine and PG545 in AsPC-1/GR and BxPC-3/GR cell lines was calculated using Calcusyn software.

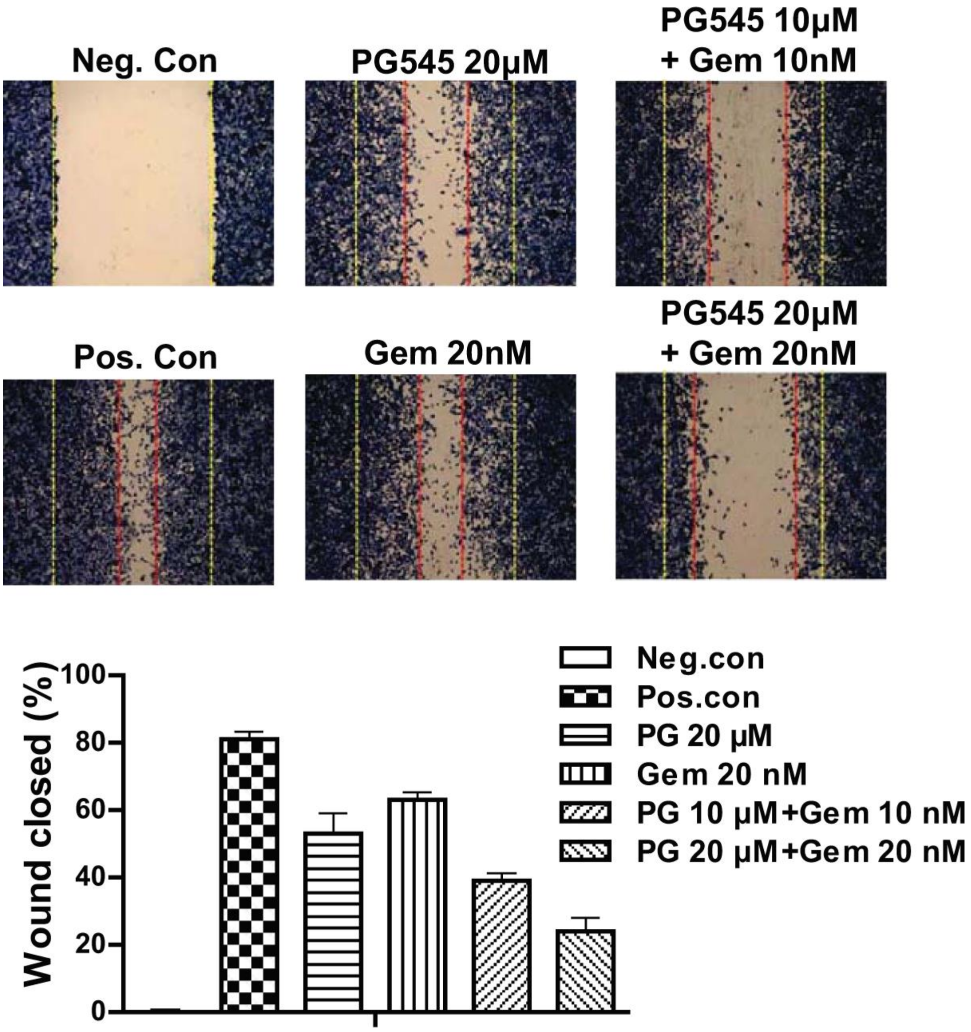

Figure S5: Combination effect of PG545 and gemcitabine on migration of AsPC-1 cells.

AsPC-1 cells were scratched and then treated with PG545 and/or gemcitabine for 48 h. The number of cells that had migrated into the scratched area was expressed as a percentage of the scratch (wound) area. Data represent means ± S.D. from three independent experiments.

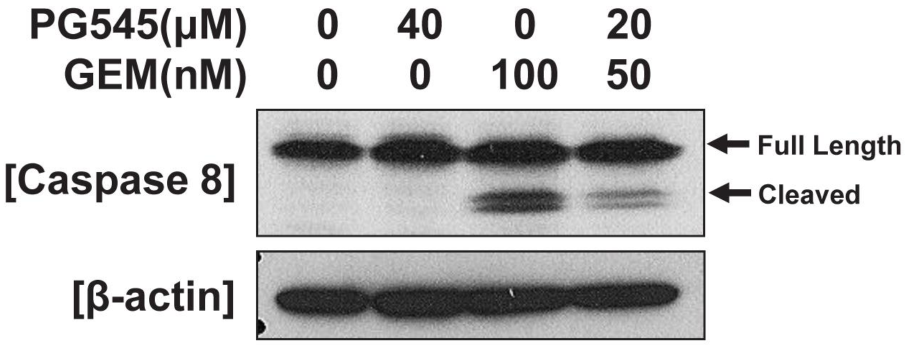

Figure S6: Effect of PG545 and gemcitabine on Caspase 8 activation.

AsPC-1 cells were treated with indicated concentrations of PG545 and gemcitabine for 48h. Western blot analysis was performed using antibodies against caspase 8 and β-actin.

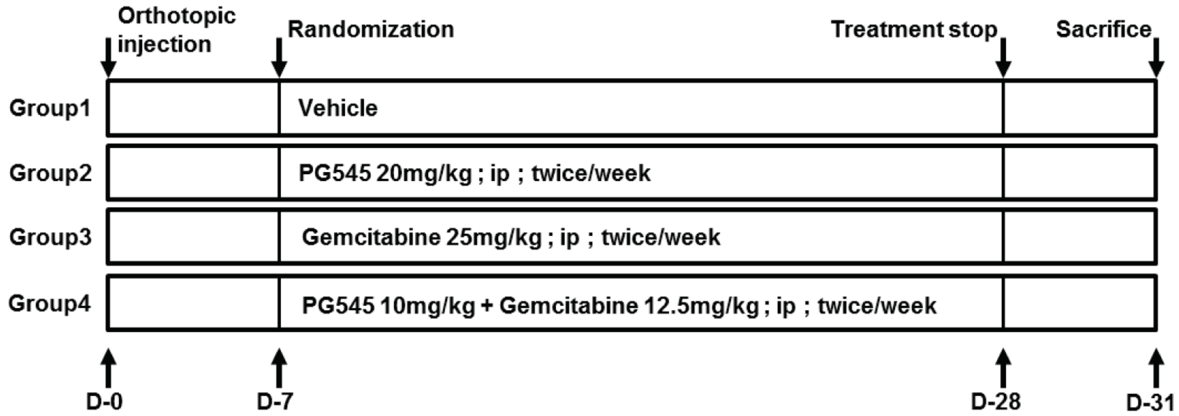

Figure S7: Schematic of experimental protocol of AsPC-1 orthotopic xenograft model.

Treatment details given in Materials and Methods. Group1 was given PBS (100 μL, twice weekly by i.p.), group2 was given PG545 (200 mg/kg, twice weekly by i.p. injection), group3 was given gemcitabine (25 mg/kg, twice weekly by i.p. injection) and group4 was given PG545 (10 mg/kg) and gemcitabine (12.5 mg/kg) twice weekly by i.p. injection.

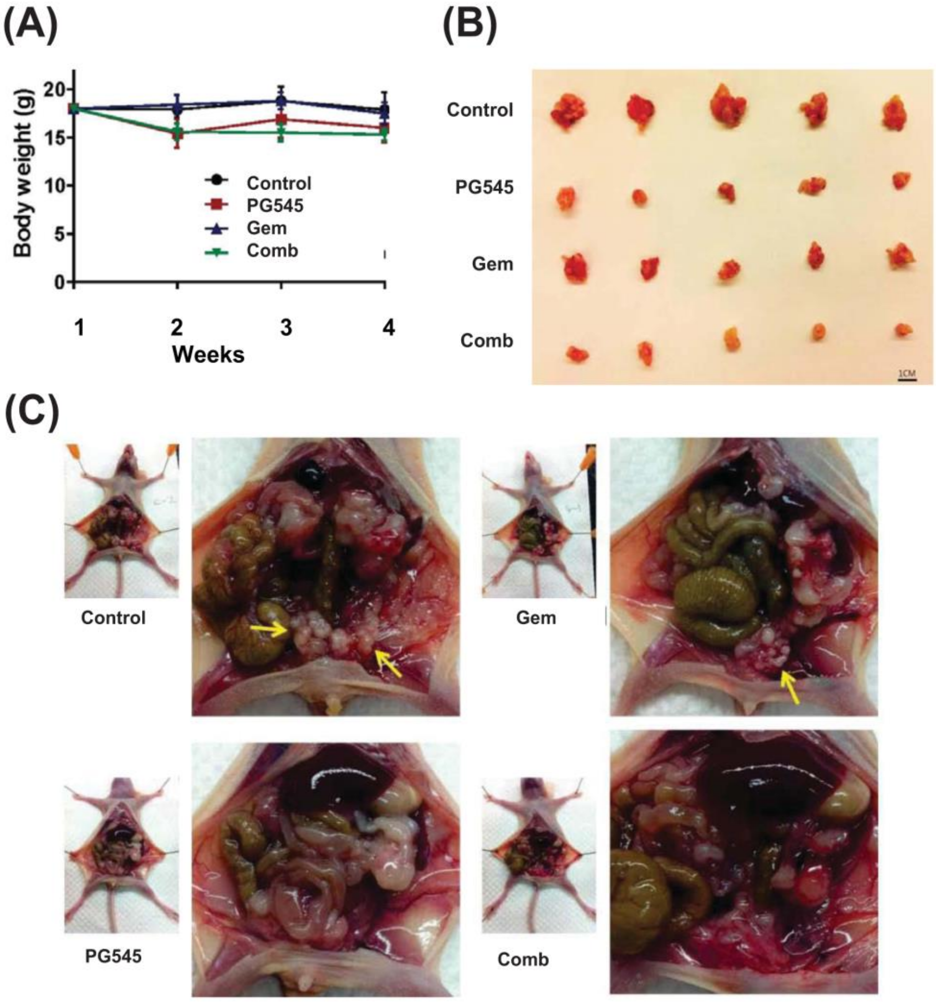

Figure S8: PG545 inhibits primary tumor growth and metastasis in AsPC-1 orthotopic xenograft mouse model.

See Materials and Methods for treatment details. (A) Mice body weights were measured each week (n = 8). Points, mean; bars, SEM. (B) Photographs of dissected tumors at completion of study. Bar 1 cm. (C) The peritoneal nodules (yellow arrows marked) were markedly suppressed in PG545 and combination treated group, compared with control or gemcitabine treated group.

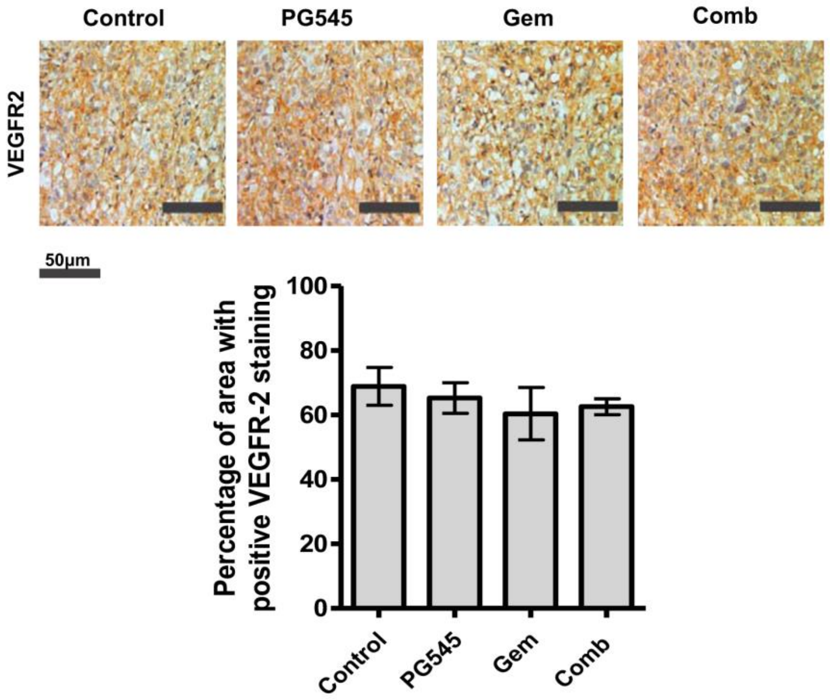

Figure S9: VEGFR2 levels in tumors from AsPC-1 orthotopic xenograft model.

Representative examples of immunohistochemical staining for VEGFR2 in tumor sections with a histogram showing quantitation of staining. Scale bars, 50 µm.

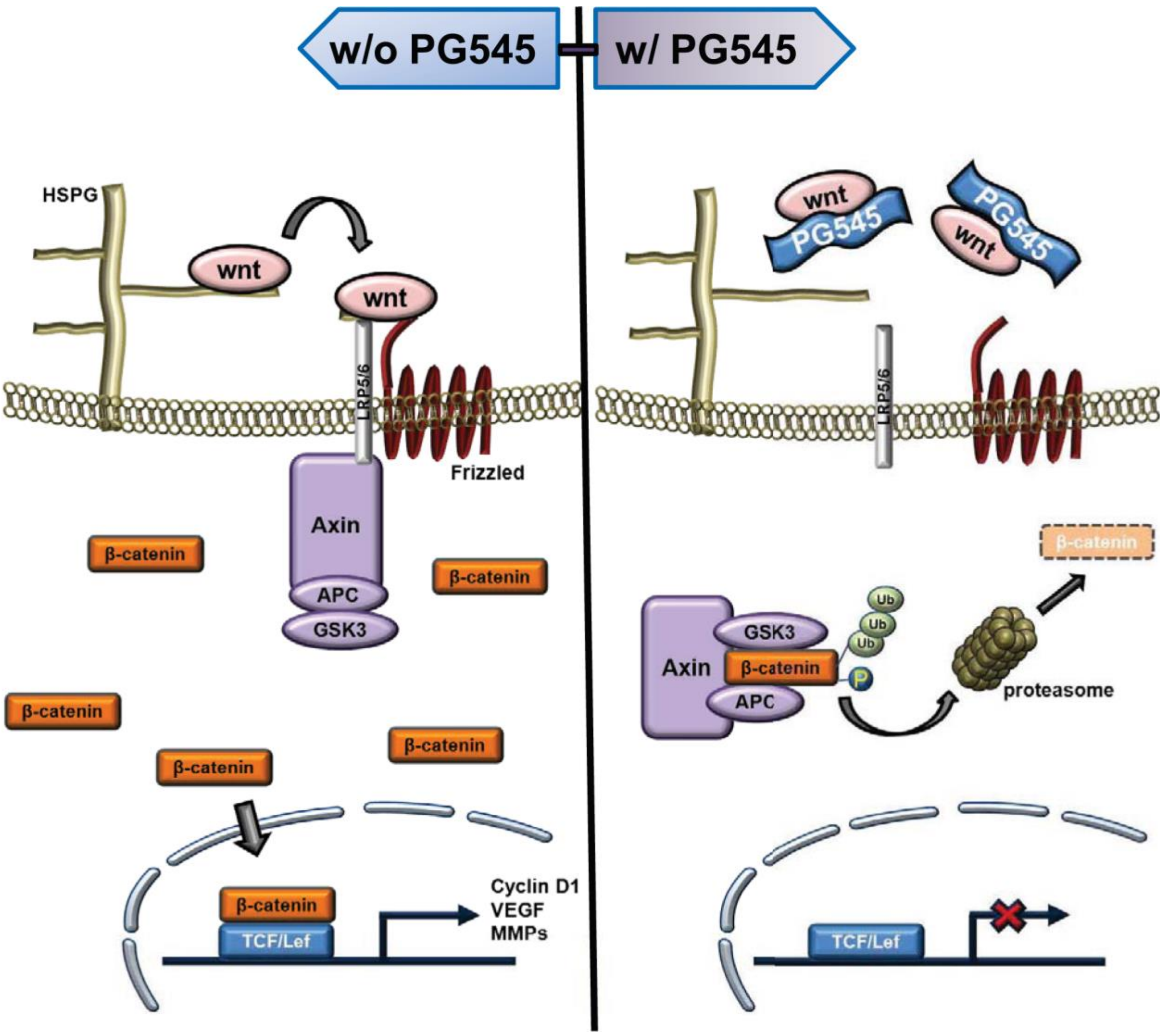

Figure S10: A model for the regulation of Wnt/β-catenin signaling by PG545.

Right side and left side represents Wnt/β-catenin signaling in the condition with and without PG545, respectively. Other target genes and proteins involved in Wnt/β-catenin pathway are excluded for simplicity.

Table S1: List of primer pairs for Real-Time Quantitative RT-PCR.

Cyclin D1	5'-CTGGAGGTCTGCGAGGAACA-3'
	5'-CCTTCATCTTAGAGGCCACGAA-3'
MMP-7	5’-GTATGGGACATTCCTCTGATCC-3’
	5’-CCAATGAATGAATGAATGGATG-3’
VEGFA	5’-CTTGCCTTGCTGCTCTACC-3’
	5’-CACACAGGATGGCTTGAAG-3’
c-Myc	5’-GGACGACGAGACCTTCATCAA-3’
	5’-CCAGCTTCTCTCAGACGAGCTT-3’
GAPDH	5′-CCACTCCTCCACCTTTGAC-3′
	5′-ACCCTGTTGCTGTAGCCA-3′

List of primer pairs for Real-Time Quantitative RT-PCR
